# Mutational single nucleotide polymorphism rs198389 and demethylation promoted natriuretic peptide B gene transcription in heart failure caused by dilated cardiomyopathy

**DOI:** 10.1016/j.gendis.2024.101345

**Published:** 2024-05-31

**Authors:** Yulong Li, Mingzhi Shen, Ting Yang, Shui Yu, Jianyuan Yin, Leiming Luo, Yali Zhao, Ping Ping, Shihui Fu

**Affiliations:** a Agency for Offices Administration of Central Military Commission, Beijing 100034, China; b Department of General Practice, Hainan Hospital of Chinese People's Liberation Army General Hospital, Sanya, Hainan 572013, China; c Central Laboratory, Hainan Hospital of Chinese People's Liberation Army General Hospital, Sanya, Hainan 572013, China; d Dermatology Department, Hainan Hospital of Chinese People's Liberation Army General Hospital, Sanya, Hainan 572013, China; e Department of Critical Care, Hainan Hospital of Chinese People's Liberation Army General Hospital, Sanya, Hainan 572013, China; f Department of Geriatric Cardiology, Chinese People's Liberation Army General Hospital, Beijing 100853, China; g General Station for Drug and Instrument Supervision and Control, Joint Logistic Support Force of Chinese People's Liberation Army, Beijing 100076, China; h Department of Cardiology, Hainan Hospital of Chinese People's Liberation Army General Hospital, Hainan Geriatric Disease Clinical Medical Research Center, Hainan Branch of China Geriatric Disease Clinical Research Center, Sanya, Hainan 572013, China

B-type natriuretic peptide (BNP) system is critical to cardiovascular physiological and pathological processes, especially in the development and progression of heart failure (HF) caused by dilated cardiomyopathy (DCM-HF).^1^^,^^2^ Single nucleotide polymorphism (SNP) in the non-coding region, especially the promoter region, might correlate well with plasma BNP levels, and potentially affect the susceptibility of DCM-HF, through interacting with transcription factor and regulating natriuretic peptide B (NPPB) gene transcription.^3^^,^^4^ Meanwhile, NPPB gene methylation might promote the process of DCM-HF development by dysregulating its gene transcription and affecting plasma BNP levels.^5^ This study demonstrated that: i) in all three SNP sites identified in the NPPB gene, mutational rs198389 in the promoter region promoted NPPB gene transcription through its combination with androgen receptor (AR); ii) as the critical transcription factor of NPPB gene, glucocorticoid receptor alpha (GR-ɑ) more obviously promoted NPPB gene transcription in combination with wild-type rs198389, and retinoid X receptor alpha (RXR-ɑ) more obviously inhibited NPPB gene transcription in combination with mutational rs198389; iii) NPPB gene methylation depressed NPPB gene transcription, and its demethylation accelerated NPPB gene unmethylation and transcription.

AR, RXR-ɑ, and GR-ɑ were cloned into the vector pcDNA3.1 and transferred into human embryonic kidney 293T (HEK293T) cells. After these cells were lysed, their supernatant was applied to analyze luciferase activity with dual-luciferase reporter assay (Promega, Madison, WI, USA) on the luminescence detector Lux-T020 (BLT, Guangzhou, China). Genomic deoxyribonucleic acid (DNA) was converted with sodium bisulfite of EZ DNA Methylation-Gold Kit (Zymo, Irvine, CA, USA) and amplified on the Applied Biosystems Veriti-384 Polymerase Chain Reaction (PCR) instrument (Thermo Fisher Scientific, Waltham, MA, USA). As shown in Figure 1A, one CpG island and two amplicons were identified between −5000 and +1000 base pairs around the transcriptional start site of NPPB gene. There were 34 and 25 CpG sites in the two amplicons of NPPB gene, respectively. EpiTYPER DNA methylation analysis (Agena, San Diego, CA, USA) was applied for quantitative analyses of NPPB gene methylation on the MassARRAY analyzer 4-matrix-assisted laser-desorption/ionization time-of-flight mass spectrometer. This study was approved by the Ethics Committee of the Chinese People's Liberation Army General Hospital (No. S2019-066-01).

**Figure 1:**
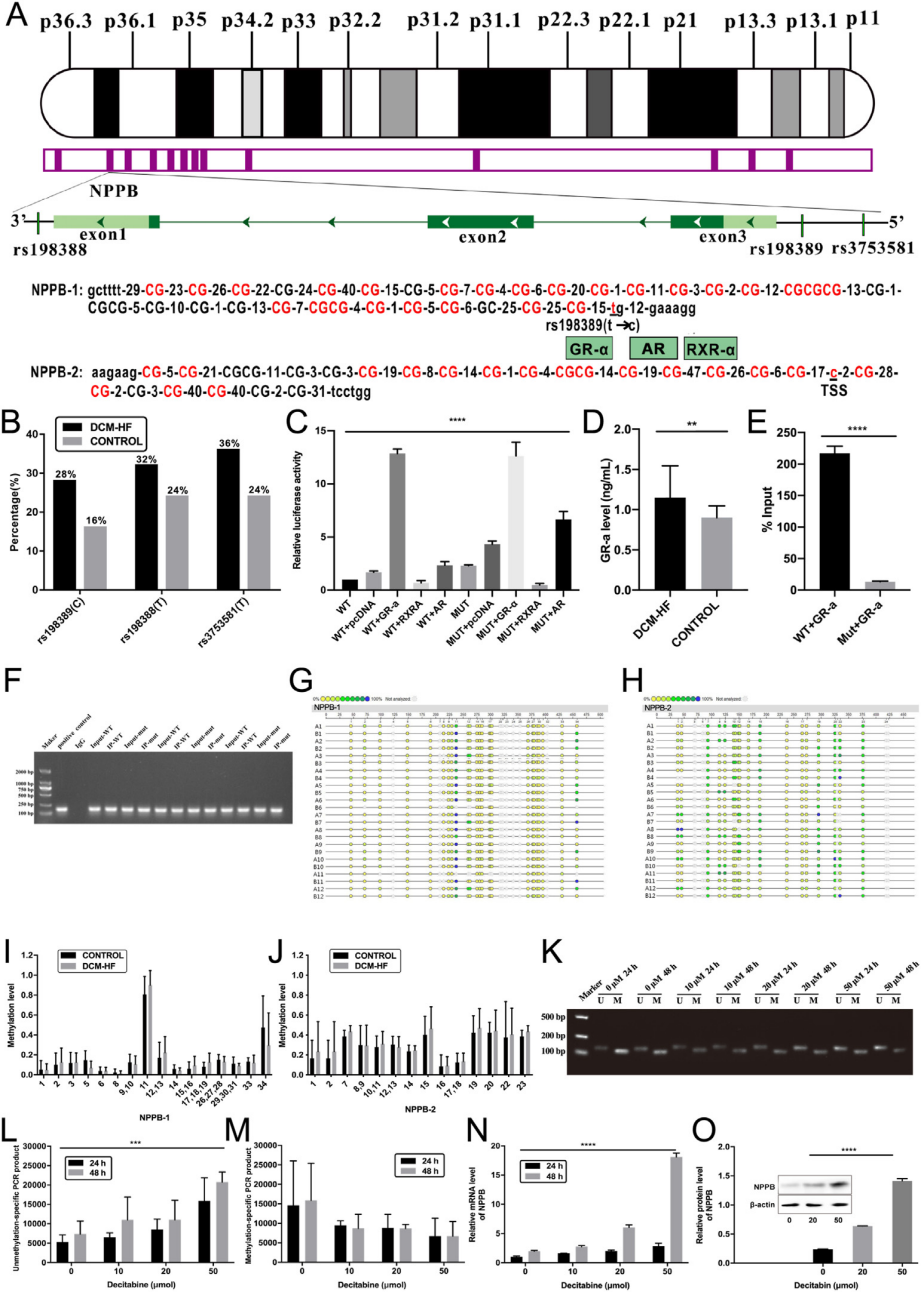
Mutational single nucleotide polymorphism (SNP) rs198389 and demethylation promoted natriuretic peptide B (NPPB) transcription in heart failure (HF) caused by dilated cardiomyopathy (DCM-HF). **(A)** Location of SNP rs198389 (T > C), critical transcription factor [androgen receptor (AR), glucocorticoid receptor alpha (GR-ɑ), and retinoid X receptor alpha (RXR-ɑ)], and 34 and 25 CpG sites in the two amplicons around the transcriptional start site (TSS) of NPPB gene. **(B)** Percentages of mutational rs198389 (C), rs198388 (T), and rs3753581 (T) in patients with HF caused by DCM-HF and healthy control participants. **(C)** Luciferase activity of human embryonic kidney 293T cells with NPPB gene promoter with wild-type (T) or mutational (C) rs198389, and with and without AR, GR-ɑ, and RXR-ɑ. **(D)** Enzyme-linked immunosorbent assay showed that GR-ɑ levels were significantly higher in patients with HF caused by DCM-HF than in healthy control participants. **(E)** Chromatin immunoprecipitation (ChIP) assay showed that GR-ɑ was more binding to NPPB gene promoter with wild-type (T) than that with mutational (C) rs198389. **(F)** ChIP assay showed that GR-ɑ was binding to both NPPB gene promoter with wild-type (T) and mutational (C) rs198389. **(G)** Matrix-assisted laser-desorption/ionization time-of-flight mass spectrometer (MALDI-TOF-MS) showed methylation levels for different CpG sites of NPPB gene-1 in patients with HF caused by DCM-HF and healthy control participants. **(H)** MALDI-TOF-MS showed methylation levels for different CpG sites of NPPB-2 in patients with DCM-HF and healthy control participants. **(I)** Methylation levels in the histogram for different CpG sites of NPPB-1 in patients with DCM-HF and healthy control participants. **(J)** Methylation levels in the histogram for different CpG sites of NPPB-2 in patients with DCM-HF and healthy control participants. **(K)** Effects of decitabine concentration and time on unmethylation-specific (U) and methylation-specific (M) Polymerase Chain Reaction (PCR) products of NPPB gene in the human cardiac myocytes (HCM). **(L)** Unmethylation-specific (U) PCR products of NPPB gene in the HCM cells were shown in the histogram. **(M)** Methylation-specific (M) PCR products of NPPB gene in the HCM cells were shown in the histogram. **(N)** Reverse transcription PCR showed that decitabine had a significant and positive relationship with messenger ribonucleic acid (mRNA) levels of NPPB gene in the HCM cells. **(O)** Western blotting showed that decitabine had a significant and positive relationship with NPPB gene protein levels in the HCM cells.

In 50 participants, including 25 patients with DCM-HF [mean age: 52 ± 11.7 years; 19 males (76.0%)] and 25 healthy control participants [mean age: 57 ± 8.8 years; 15 males (60.0%); *p* > 0.05 for all]. Sanger sequence showed no SNP sites in its three exons and two introns. Three SNP sites were identified in other regions, including rs198389 (T > C) in the promoter region, rs198388 (C > T) in the 3′ non-coding region, and rs3753581 (C > T) in the 5′ non-coding region (Fig. 1A). Percentages of mutational rs198389 (C), rs198388 (T), and rs3753581 (T) had no difference between patients with DCM-HF and healthy control participants (*P* > 0.05 for all; Fig. 1B). Mutational rs198389 (C) (*r* = 0.289, *P* < 0.05) rather than rs3753581 (T) (*r* = 0.265, *P* > 0.05) and rs198388 (T) (*r* = 0.213, *P* > 0.05) positively correlated with NT-proBNP levels. Luciferase activity of HEK293T cells with NPPB gene promoter with mutational rs198389 (C) was significantly higher than that of HEK293T cells with NPPB gene promoter with wild-type rs198389 (T) (*P* < 0.05; Fig. 1C), suggesting that mutational rs198389 promoted NPPB gene transcription.

Whether there was NPPB gene promoter with wild-type (T) or mutational (C) rs198389, luciferase activity of HEK293T cells was significantly increased after the overexpression of AR and GR-ɑ and decreased after the overexpression of RXR-ɑ (*P* < 0.05 for all; Fig. 1C). When with NPPB gene promoter with wild-type rs198389 (T), increased luciferase activity of HEK293T cells induced by AR and decreased luciferase activity of HEK293T cells induced by RXR-ɑ were significantly less and increased luciferase activity of HEK293T cells induced by GR-ɑ was significantly more than those of HEK293T cells with NPPB gene promoter with mutational rs198389 (C) (*P* < 0.05 for all), suggesting that mutational rs198389 increased promotion effect of AR and inhibition effect of RXR-ɑ and reduced promotion effect of GR-ɑ on NPPB gene transcription. In 70 participants, including 35 patients with DCM-HF [mean age: 54 ± 14.3 years; 29 males (82.9%)] and 35 healthy control participants [mean age: 61 ± 12.3 years; 24 males (68.6%); *P* > 0.05 for all], enzyme-linked immunosorbent assay showed that GR-ɑ levels were significantly higher in patients with DCM-HF than healthy control participants (*P* < 0.05; Fig. 1D). GR-ɑ levels positively correlated with NT-proBNP levels (*r* = 0.318, *P* < 0.05). Chromatin immunoprecipitation assay showed that GR-ɑ was binding to both NPPB gene promoter with wild-type (T) and mutational (C) rs198389, and more binding to NPPB gene promoter with wild-type (T) than that with mutational (C) rs198389 (*P* < 0.05; Fig. 1E, F), suggesting that GR-ɑ promoted NPPB gene transcription in combination with wild-type rs198389.

In 24 participants, including 12 patients with DCM-HF [mean age: 55 ± 10.0 years; 10 males (83.3%)] and 12 healthy control participants [mean age: 57 ± 11.9 years; 8 males (66.7%); *P* > 0.05 for all], Figure 1G–J showed methylation levels for different CpG sites of NPPB gene in patients with DCM-HF and healthy control participants. The cpG_5 site of NPPB gene was significantly hypo-methylated in patients with DCM-HF compared with healthy control participants (*P* < 0.05). Its methylation levels negatively correlated with NT-proBNP levels (*r* = −0.428, *P* < 0.05).

There were both methylation-specific and unmethylation-specific PCR products of NPPB gene in the human cardiac myocytes (HCM) cells (Fig. 1K−M). Unmethylation-specific PCR products had a gradual increment in the HCM cells as the concentrations of decitabine increased from 0 to 50 μM, showing a significantly positive relationship between decitabine concentrations and gene unmethylation (*P* < 0.05). At the same concentrations of decitabine, unmethylation-specific PCR products treated for 48 h were more than those treated for 24 h, showing a significant concentration-time dependence (*P* < 0.05). NPPB gene messenger ribonucleic acid (mRNA) levels were gradually elevated in the HCM cells as the concentrations of decitabine increased from 0 to 50 μM, showing a significantly positive relationship between decitabine concentrations and gene transcription (*P* < 0.05; Fig. 1N). At the same concentrations of decitabine, NPPB gene mRNA levels treated for 48 h were higher than those treated for 24 h, showing a significant concentration-time dependence (*P* < 0.05). NPPB gene protein levels were gradually up-regulated in the HCM cells as the concentrations of decitabine increased from 0 to 50 μM, showing a significantly positive relationship between decitabine concentrations and gene translation (*P* < 0.05; Fig. 1O).

Mutational rs198389 has been illustrated to be very common and related to higher BNP levels in the general US population.^2^, ^3^, ^4^ However, there have been nearly no basic studies to confirm realistic roles and molecule mechanisms of SNP profoundly regulating NPPB gene transcription. This study confirmed that mutational rs198389 promoted NPPB gene transcription through its combination with AR. GR-ɑ more obviously promoted NPPB gene transcription in combination with wild-type rs198389, and RXR-ɑ more obviously inhibited NPPB gene transcription in combination with mutational rs198389.

It remains unclear whether NPPB gene hyper-methylation leads to remarkably reduced NPPB gene transcription, and demethylation induces changed methylation and transcription of NPPB gene.^5^ Firstly, this study found that NPPB gene transcription was down-regulated owing to DNA methylation. Secondly, this study realized that DNA methyltransferase inhibitors resolved methylative suppression of NPPB gene and effectively promoted its subsequent transcription.

This study supported mutational rs198389, transcription factor, and methylation as the significant mechanisms regulating NPPB gene transcription in DCM-HF might enjoy therapeutic strategies based on gene editing, transcription factor enhancement, and demethylation of NPPB gene. Future population-based studies with large-scale samples could be performed to analyze their effects on disease severity or patient prognosis of DCM-HF.

## Ethics declaration

This study was approved by the Ethics Committee of the Chinese People's Liberation Army General Hospital (No. S2019-066-01).

## Author contributions

All authors contributed to the study design, conducted the data collection and analyses, and drafted the manuscript.

## Data availability

The data will be available from the corresponding author upon reasonable request.

## Conflict of interests

The authors declared no competing interests.

## Funding

This work was supported by grants from the Excellent Youth Incubation Program of Chinese People's Liberation Army General Hospital (No. 2020-YQPY-007), the 10.13039/501100004761Natural Science Foundation of Hainan Province, China (No. 821QN389, 821MS117, 823MS161, 820MS124, 821MS112, 822MS198, 820MS126, 820QN383, 822MS193), the Military Medical Science and Technology Youth Incubation Program (China) (No. 20QNPY110, 19QNPY060), the Key R&D Program of Hainan Province, China (No. ZDYF2023SHFZ145), the Health Care Project of PLA (China) (No. 22BJZ30), the Clinical Medical Research Center Project of Hainan Province, China (LCYX202106, LCYX202201, LCYX202303), the 10.13039/501100012166National Key R&D Program of China (No. 2018YFC2000400), the National S&T Resource Sharing Service Platform Project of China (No. YCZYPT[2018]07), the Specific Research Fund of Innovation Platform for Academicians of Hainan Province, China (No. YSPTZX202216), the Heatstroke Treatment and Research Center of PLA (China) (No. 413EGZ1D10), and the Major Science and Technology Programme of Hainan Province, China (No. ZDKJ2019012). The sponsors had no role in the design, conduct, interpretation, review, approval, or control of this article.
