# Supplementary material for: Mutational single nucleotide polymorphism rs198389 and demethylation promoted natriuretic peptide B gene transcription in heart failure caused by dilated cardiomyopathy
Source: Genes Dis. 2024 May 31;12(2):101345. doi: 10.1016/j.gendis.2024.101345 (PMC11625330; doi:10.1016/j.gendis.2024.101345)
Supplement: Multimedia component 1 [file mmc1.doc]

**BACKGROUND**

Heart failure (HF) remains an enormous public health problem despite many advances in its diagnosis and treatment in the recent decades, with a prevalence of 5.7 million individuals and an incidence of 500000 new cases annually^1^. There is an urgent need for precise diagnosis and treatment to prevent the development of HF and improve the prognosis of patients with HF. Cardiac ventricles mainly produce B-type natriuretic peptide (BNP), which is increased in the failing heart in proportion to the severity of HF^2^. BNP system is critical to cardiovascular physiological and pathological processes, especially in the development and progression of HF caused by dilated cardiomyopathy (DCM-HF), and has been widely applied as a valuable tool in the diagnostic determination, therapeutic management and prognostic evaluation of patients with DCM-HF. As a god-given and beneficial system, BNP system promotes natural counterbalance of renin-angiotensin and sympathetic nervous systems^3^. The change in the BNP system and its roles might be genetic in origin, considering significant relationships between genetic variants and cardiac states^4^. Single nucleotide polymorphism (SNP) in the coding region (exons) of natriuretic peptide B (NPPB) gene might affect the production and degradation of BNP, and cause the production of BNP truncated forms^5^. SNP in the non-coding region, including introns, upstream and downstream gene, especially promoter region, might also correlate well with plasma BNP levels and function, and potentially affect the susceptibility and prognosis of DCM-HF, through regulating NPPB gene transcription with the help of transcription factor (TF)^6^. Several candidate SNP sites affecting BNP levels were identified in previous epidemiological association studies^7^. However, considering inconsistency results commonly existed in these studies, it is very necessary to preform basic molecule studies to confirm realistic roles and molecule mechanisms of SNP profoundly regulating NPPB gene transcription. Meanwhile, gene methylation, a kind of epigenetic mechanism, might be inherited or result from somatic changes^8,9^. It has been suggested in recent studies that gene methylation was involved in dysregulated gene transcription, especially the hypo-methylation or hyper-methylation of CpG islands in the promoter region could lead to up-regulated or down-regulated gene transcription in various cancers and other diseases^10,11^. Thus, NPPB gene methylation might promote the process of DCM-HF development through dysregulating its gene transcription and affecting plasma BNP levels. As the first time worldwide, the present study aimed to explore unknown roles of SNP, TF and methylation regulating NPPB gene transcription in DCM-HF.

**METHODS**

**Study patients**

All participants were older than 18 years and recruited from Cardiology Department of Chinese People's Liberation Army General Hospital. According to European Society of Cardiology Guidelines for the diagnosis and treatment of HF, HF was diagnosed by chief physician on the basis of medical history, physical examination, blood test and instrumental result. According to Chinese Guidelines for the diagnosis and treatment of DCM, DCM was diagnosed by chief physician based on left ventricular dilation and impaired left ventricular systolic function. Coronary angiography was performed to distinguish DCM and coronary artery disease. Exclusion criterion: 1) severe aortic stenosis; 2). left ventricular auxiliary device; 3) heart transplant; 4). malignant tumor; 5) immunodeficiency; 6) severe infection; 7) mental disorder; and 8) drug abuse. Baseline characteristics, such as age and sex, had no difference between patients with DCM-HF and healthy control participants in all analyses (P>0.05 for all).

### SNP analysis

### NPPB gene (NC_00000 1.11) located in the chr1:11857464-11858945, which encode precursor form of BNP, including three exons, two introns, 5' and 3' non-coding regions. For SNP analysis, the present study enrolled 50 participants, including 25 patients with DCM-HF [mean age: 52±11.7 years; 19 males (76.0%)] and 25 healthy control participants [mean age: 57±8.8 years; 15 males (60.0%)]. Samples of venous blood were extracted from participants between 6am and 7am. In order to identify the SNP sites of NPPB gene, Sanger Sequence was preceded by genomic deoxyribonucleic acid (DNA) from blood leukocytes by applying TIANamp Blood DNA Kit (TIANGEN, Beijing, China). Primers were designed using the software of Primer Premier 3.0 (Premier, Palo Alto, CA, USA) and synthesized at the Beijing Genomics Institute (BGI, Shenzhen, China). Polymerase Chain Reaction (PCR) was performed using an enzymatic method (Ex Taq DNA polymerase, [TAKARA,](https://www.sogou.com/link?url=DSOYnZeCC_odm7_-pldo3jfPuU0JBiutG_H1iKm2_78.) Shiga, Japan) on the PTC-200 PCR (Bio-Rad, Hercules, CA, USA). Agarose gel electrophoresis was applied to detect PCR products on the DYY-8C Electrophoresis Power Supply (Liuyi, Beijing, China). PCR products were purified using 96 Well Plate for DNA Purification (Merck Millipore, Darmstadt, Germany) according to the protocol provided by the manufacturer. DNA fragment was applied to conduct minisequencing reaction using Bigdye v3.1 Cycle Sequencing Kit on the Applied Biosystems 3730XL Genetic Analyser (Thermo Fisher Scientific, Waltham, MA, USA).

### Luciferase activity

### Human embryonic kidney 293T (HEK293T) cells were maintained in the dulbecco's modified eagle medium ([high glucose,](https://www.sogou.com/link?url=hedJjaC291P6DaRYPNQxHSZUGlA023BniqnsupH2ZMaPWQKsnl3STcNC5d7lS29jdPkQFW5yNY1W5FAx3ud4oQ..) GIBCO, Thermo Fisher Scientific, Waltham, MA, USA) and supplemented with fetal bovine serum (GIBCO, Thermo Fisher Scientific, Waltham, MA, USA) at 37℃ in the humidified atmosphere with 5% CO_2_. NPPB gene promoters with wild-type (T) and mutational (C) rs198389 were cloned into the vector psicheck2 produced by GenScript (New Jersey, USA). PROMO version 3.0.2 was applied as bioinformatics analysis to identify the TF binding to NPPB gene. Androgen receptor (AR), [retinoid X receptor alpha](https://www.ncbi.nlm.nih.gov/gene/6256) (RXR-ɑ) and glucocorticoid receptor alpha (GR-ɑ) were identified to be the critical TF of NPPB gene with different promoters, including wild-type (T) or mutational (C) rs198389. AR, RXR-ɑ and GR-ɑ genes were cloned into the vector pcDNA3.1 and transferred into HEK293T cells using transfection reagents (Hanbio, Shanghai, China). After these HEK293T cells were lysed, their supernatant was applied to analyze luciferase activity (relative light unit) with Dual-Luciferase Reporter Assay System (Promega, Madison, WI, USA) on the luminescence detector Lux-T020 (BLT, Guangzhou, China).

### **ELISA analysis**

### For enzyme-linked immunosorbent assay (ELISA) analysis, the present study enrolled 70 participants, including 35 patients with DCM-HF [mean age: 54±14.3 years; 29 males (82.9%)] and 35 healthy control participants [mean age: 61±12.3 years; 24 males (68.6%)]. Plasma levels of GR-ɑ were determined by Human GR-ɑ Enzyme-linked Immunosorbent Assay Kit (Elabscience, Wuhan, China).

### ChIP assay

### Chromatin Immunoprecipitation (ChIP) assay was performed following the primers (CTGCCTTTTCCAGCAACGGT, TATAGAAATGGGCCGGGTCG) using digested chromatin from HEK293T cells with Anti-GR antibody (ChIP Grade,​Abcam, Cambridge, UK), Histone H3 (D2B12) XP Rabbit mAb (Positive control) and Normal Rabbit immunoglobulin G (Negative control) according to the protocol of SimpleChIP® Enzymatic Chromatin IP Kit (ChIP-Grade Protein G Magnetic Beads, Cell Signaling Technology, Danvers, MA, USA). Purified DNA was analyzed by standard PCR methods on the PCR instrument (Donglin, Beijing, China). The amount of immunoprecipitated DNA in each sample is represented as signal relative to the amount of input chromatin.

**Methylation analysis**

For methylation analysis, there were 24 participants, including 12 patients with DCM-HF [mean age: 55±10.0 years; 10 males (83.3%)] and 12 healthy control participants [mean age: 57±11.9 years; 8 males (66.7%)]. Participants provided venous blood from which genomic DNA was extracted for methylation analysis of NPPB gene. Magnetic Beads Blood DNA Extraction Midi Kit III (BioTeKe, Beijing, China) was applied for DNA extraction on the Automated Nucleic Acid Extraction System (BioTeKe, Beijing, China). Quality and quantity of DNA in all samples were evaluated by agarose gel electrophoresis and NanoDrop 2000 spectrophotometer (Thermo Fisher Scientific, Waltham, MA, USA). Agarose gel electrophoresis showed that there were visible bands and no degradation of DNA in all samples. NanoDrop 2000 spectrophotometer showed that DNA concentrations were higher than 30ng/µl and total quantities were higher than 1µg in these samples. Meanwhile, OD260/280 values were between 1.5 and 2.0, and OD260/230 values were higher than 0.6. Genomic DNA from each sample was converted with sodium bisulfite of EZ DNA Methylation-Gold Kit (Zymo, Irvine, CA, USA) and amplified on the Applied Biosystems Veriti-384 PCR instrument (Thermo Fisher Scientific, Waltham, MA, USA). Unmethylated cytosine was converted to uracil and subsequently to thymine during the PCR. Conversely, methylated cytosine was not converted and allowed the assessment of methylated levels. As shown in Figure 1A, one CpG island and two amplicons were identified between -5000~+1000 base pairs around the transcriptional start site (TSS) of NPPB gene. Altogether, there were 34 and 25 CpG sites in the two amplicons of NPPB gene, respectively. Agena EpiDesigner (Agena, San Diego, CA, USA) was applied to obtain the following two pairs of primers on the basis of its reverse complemented strand (aggaagagagTTTTTAATGAATTGTTATTGGGGAA, cagtaatacgactcactatagggagaaggctTCTTCCTTTCCTACAAATATCCAAA) and (aggaagagagTTTGGATATTTGTAGGAAAGGAAGA, cagtaatacgactcactatagggagaaggctCCAAAAAAACCAAATACAAAAAAAA) to amplify base pairs. An additional T7 promoter tag (5’-cagtaatacgactcactatagggagaaggct-3’) for in vitro transcription was added for each reverse primer. A 10-mer tag (5’-aggaagagag-3’) was added on each forward primer to adjust for melting-temperature differences between forward and reverse primers. T7 Transcription/RNase A Cocktail was applied to achieve ribonucleic acid (RNA) base-specific cleavage and produce unmethylated and methylated CpG containing fragments that were resolved and quantified for methylation levels. EpiTYPER DNA Methylation Analysis Technology (Agena, San Diego, CA, USA) was applied for quantitative analyses of NPPB gene methylation with EpiTYPER Reagent Kit on the MassARRAY Analyzer 4-Matrix-Assisted Laser-Desorption/Ionization Time-Of-Flight Mass Spectrometer (MALDI-TOF-MS). Methylation levels for CpG sites were generated by EpiTYPER Reporting Software (Agena, San Diego, CA, USA), and compared between patients with DCM-HF and healthy control participants.

**Methylation-specific PCR**

Human Cardiac Myocytes (HCM) cells were maintained in the dulbecco's modified eagle medium ([high glucose,](https://www.sogou.com/link?url=hedJjaC291P6DaRYPNQxHSZUGlA023BniqnsupH2ZMaPWQKsnl3STcNC5d7lS29jdPkQFW5yNY1W5FAx3ud4oQ..) GIBCO, Thermo Fisher Scientific, Waltham, MA, USA) and supplemented with fetal bovine serum (GIBCO, Thermo Fisher Scientific, Waltham, MA, USA) at 37℃ in the humidified atmosphere with 5% CO_2_. After cell culture for 24 hours, HCM cells were treated with 0, 10, 20 and 50μM of DNA methyltransferase inhibitor, decitabine (Selleck, Houston, Texas, USA), and cultivated for 24 and 48 hours, respectively. Genomic DNA was extracted from HCM cells using TIANamp Genomic DNA Kit (TIANGEN, Beijing, China), treated with sodium bisulfite (Sinopharm, Shanghai, China) and amplified with TaKaRa EpiTaq HS for bisulfite-treated DNA (Kusatsu, Shiga, Japan) on the PCR instrument (Donglin, Beijing, China). There were unmethylation-specific primers (Forward: TTTTAGGATAAAAGGTTATGG; Reverse: TACTACTACTACTACTACAATACATCCAAA) and methylation-specific primers (Forward: GTATGGTAGGGTAGGTTCGATA; Reverse: ACTACTACGATACGTCCGAA) applied in the methylation-specific PCR, respectively. PCR products were detected with agarose gel electrophoresis and observed with Tanon 1600 Automated Digital Gel Image Analysis System (Tanon, Shanghai, China). Grayscale analysis of images was performed with Image J software.

**RNA qualification**

Total RNA was extracted from HCM cells using TRIzol Reagent (Invitrogen, Thermo Fisher Scientific, Waltham, MA, USA). Purity and concentrations of RNA in all samples were ascertained by Nano 200 ultra low volume spectrophotometer (Allsheng, Hangzhou, China). Reverse Transcription (RT)-PCR was performed on Applied Biosystems Real-time Fluorescence Quantitative PCR instrument (Thermo Fisher Scientific, Waltham, MA, USA) to quantify messenger ribonucleic acid (mRNA) levels after normalizing with glyceraldehyde-phosphate dehydrogenase (GAPDH), which served as an endogenous control. There were NPPB primers (Forward: GCTCCTGCTCTTCTTGCATC; Reverse: GGACTTCCAGACACCTGTGG) and GAPDH primers (Forward: CCAGGTGGTCTCCTCTGA; Reverse: GCTGTAGCCAAATCGTTGT) applied in the PCR, respectively. The ΔΔCT method was applied to calculate CT value for each sample, and the results were expressed as 2^ΔΔCT^ to represent mRNA levels of NPPB gene: ΔΔCT = (CTother concentrations of decitabine for 24 and 48 hours - CT_GAPDH_) - (CT _0μM of decitabine for 24 hours_ - CT_GAPDH_).

**Western blotting**

HCM cells treated with decitabine for 48 hours were lysed with radioimmunoprecipitation assay (RIPA) buffer ([Beyotime](https://www.sogou.com/link?url=hedJjaC291NdfGwkRuQnxbOkLkN5U8cEMDa0VX57lN5E1ZqSVbHBvvEq15QEuE-2ZOoFBdMNmH0mf5EiVB9ZLf0krIrW5HqOpAHEu7C-SRZ8zKFmb0bJUiQwpL68vLMX6BlP2CujqZk.), Shanghai, China) containing [Phenylmethanesulfonyl fluoride](http://dict.youdao.com/w/eng/pmsf_phenylmethanesulfonyl_fluoride/#keyfrom=dict.phrase.wordgroup). Concentrations of extracted proteins were quantified using Bicinchoninic acid (BCA) Protein Quantification Kit (Dingguo, Beijing, China) according to the manufacturer’s instructions. Western blotting (WB) was performed using BNP antibody (GeneTex, Irvine, CA, USA) and Anti-Rabbit IgG (H+L) Antibody (KPL, SeraCare, Milford, MA, USA) after sodium dodecyl sulfate polyacrylamide gel electrophoresis. Grayscale analysis of images was performed with Image J software.

**Statistical analysis**

Data were presented using mean and standard deviation (continuous variables with normal distribution), median and interquartile range (continuous variables with skewed distribution), and number and percentage (categorical variables). Data were compared between patients with DCM-HF and healthy control participants, using Student’s t-test for continuous variables with normal distribution, Mann–Whitney U test for continuous variables with skewed distribution, and Chi-square test for categorical variables. Effects of treated concentrations and time of decitabine on the methylation-specific and unmethylation-specific PCR products, as well as mRNA and protein levels of NPPB gene, were assessed using univariate analysis of variance (ANOVA). Pearson and Spearman correlations were applied for continuous variables with normal distribution or continuous variables with skewed distribution and categorical variables, respectively. Statistical significance was set at two-tailed P<0.05. Statistical analysis was performed using Statistic Package for Social Science (SPSS) version 17.0 (SPSS, Chicago, IL, USA) and Graphpad [Prism](https://www.sogou.com/link?url=hedJjaC291OfPyaFZYFLI4KQWvqt63NBcGkw8UDYdqWSksagzMw4zw..) version 8 (Graphpad, San Diego, CA, USA).

**Discussion**

BNP plays significant roles in the pathophysiology and development of HF, and has become diagnostic, therapeutic and prognostic tools widely applied in HF^12^. BNP system variability will continue to be a relevant research and clinical issue not only because our knowledge of BNP system biology is still very incomplete, but also because BNP is being increasingly explored towards personalized evaluation and care, and because there are numerous novel NP therapeutics currently under the development^12^. Knowledge about SNP of NPPB gene might result in better understanding of molecular metabolism and regulation of BNP and novel discovery of therapeutic targets in patients with DCM-HF^13^. SNP in the coding region (exons) of NPPB gene might correlate with the production and degradation of BNP, and potentially affect the production of BNP truncated forms^14^. The present study indicated that there was no SNP sites in the three exons and two introns of NPPB gene. However, three SNP sites were identified in other regions of NPPB gene, including rs198389 in the promoter region, rs3753581 in the 5' non-coding region, and rs198388 in the 3' non-coding region, identified in the present study.

Relationship between SNP rs198389 and BNP levels has been detected in previous epidemiological association studies. Mutational rs198389 has been illustrated to be very common and related to higher BNP levels in general US population^15^. In the Atherosclerosis Risk in Communities Study, mutational rs198389 was related to elevated NT-proBNP levels in 11361 black and white participants^16^. Meanwhile, mutational (C) rs198389 was also related to higher BNP levels in patients with coronary artery disease or undergoing elective cardiac catheterization^14^. However, these has been nearly no basic molecule studies to confirm realistic roles and molecule mechanisms of SNP profoundly regulating NPPB gene transcription. Based on the reliably data from Sanger Sequence and Dual-Luciferase Reporter Assay, the present study not only revealed positive relationship between mutational rs198389 and NT-proBNP levels, but also confirmed that mutational rs198389 promoted NPPB gene transcription through its combination with AR. Moreover, GR-ɑ and RXR-ɑ were also identified as the critical TF of NPPB gene. GR-ɑ more obviously promoted NPPB gene transcription in combination with wild-type rs198389, and RXR-ɑ more obviously inhibited NPPB gene transcription in combination with mutational rs198389.

Recently, epigenetic silencing of NPPB gene by DNA hyper-methylation has not received enough attention in a variety of cardiovascular disorders, especially in the development of DCM-HF^10^. It still remains unclear that whether NPPB gene hyper-methylation leads to remarkably reduced NPPB gene transcription, and demethylation induces changed methylation and transcription of NPPB gene^11^. Firstly, the present study assessed methylation levels of NPPB gene using MassARRAY-based quantitative methylation analysis in patients with DCM-HF and healthy control participants, and the results revealed that differential sit of NPPB gene upstream has down-regulated methylation in patients with DCM-HF. Secondly, the present study explored epigenetic mechanisms regulating NPPB gene transcription, and found that its transcription was down-regulated owing to DNA methylation. Thirdly, the present study realized that DNA methyltransferases inhibitor resolved methylative suppression of NPPB gene and effectively promoted its subsequent transcription, providing the possibility that demethylation might be applied to design optimal method for DCM-HF treatment.

**REFERENCES**

1. Virani, S. S. et al. American Heart Association Council on Epidemiology and Prevention Statistics Committee and Stroke Statistics Subcommittee. Heart Disease and Stroke Statistics-2020 Update: A Report from the American Heart Association. *Circulation.* **141**(9), e139-e596 (2020).
2. Zois, N. E. et al. Natriuretic peptides in cardiometabolic regulation and disease. *Nat Rev Cardiol*. **11**(7), 403-12 (2014).
3. Fu, S., Ping, P., Wang, F. & Luo, L. Synthesis, secretion, function, metabolism and application of natriuretic peptides in heart failure. *J Biol Eng*. **12**, 2 (2018).
4. Lanfear, D. E. Genetic variation in the natriuretic peptide system and heart failure. *Heart Fail Rev*. **15**(3), 219-228 (2010).
5. Poreba, R. et al. SNP rs198389 (T-381 C) polymorphism in the B-type natriuretic peptide gene promoter in patients with atherosclerotic renovascular hypertension. *Pol Arch Med Wewn*. **119**(4), 219-24 (2009).
6. Yandle, T. G. & Richards, A. M. B-type Natriuretic Peptide circulating forms: analytical and bioactivity issues. *Clin Chim Acta*. **448**, 195-205 (2015).
7. Tiret, L., Mallet, C. & Poirier, O. Lack of association between polymorphisms of eight candidate genes and idiopathic dilated cardiomyopathy: the CARDIGENE study. *J Am Coll Cardiol*. **35**(1), 29-35 (2000).
8. Lim, U. & Song, M. A. Dietary and lifestyle factors of DNA methylation. Methods Mol Biol. 863, 359-376 (2012).
9. Feinberg, A. P. Phenotypic plasticity and the epigenetics of human disease. *Nature*. **447**, 433-440 (2007).
10. Lopez-Serra, P. & Esteller, M. DNA methylation-associated silencing of tumor-suppressor microRNAs in cancer. *Oncogene*. **31**(13), 1609-22 (2012).
11. Thomson, J. P. et al. CpG islands influence chromatin structure via the CpG-binding protein Cfp1. *Nature*. **464**, 1082-1086 (2010).
12. Fu, S., Ping, P., Zhu, Q., Ye, P. & Luo, L. Brain Natriuretic Peptide and Its Biochemical, Analytical, and Clinical Issues in Heart Failure: A Narrative Review. *Front Physiol*. **9**, 692 (2018).
13. Fox, A. A., Collard, C. D. & Shernan, S. K. Natriuretic peptide system gene variants are associated with ventricular dysfunction after coronary artery bypass grafting. *Anesthesiology*. **110**(4), 738-747 (2009).
14. Lanfear, D. E., Stolker, J. M. & Marsh, S. Genetic variation in the B-type natriuretic peptide pathway affects BNP levels. *Cardiovasc Drugs Ther*. **21**(1), 55-62 (2007).
15. Costello-Boerrigter, L. C., Boerrigter, G. & Ameenuddin, S. The effect of the brain-type natriuretic peptide single-nucleotide polymorphism rs198389 on test characteristics of common assays. *Mayo Clin Proc*. **86**(3), 210-218 (2011).
16. Fu S, Jiao J, Guo Y, Zhu B, Luo L. N-terminal pro-brain natriuretic peptide levels had an independent and added ability in the evaluation of all-cause mortality in older Chinese patients with atrial fibrillation. *BMC Geriatr.* **19**(1), 56 (2019).
